# Forcing your luck: Goal-striving behavior in chance situations

**DOI:** 10.1007/s11031-015-9527-5

**Published:** 2015-12-09

**Authors:** Daniela Becker, Joop van der Pligt

**Affiliations:** Department of Social Psychology, University of Amsterdam, Weesperplein 4, 1018 XA Amsterdam, The Netherlands

**Keywords:** Automatic goal pursuit, Goal-gradient, Illusion of control, Resource conservation, Uncertainty

## Abstract

Previous research suggests that desired end-states (i.e., goals) initiate a set of motivational processes supporting goal-attainment. For example, motivational intensity (e.g., effort investment) increases as distance to the goal decreases. The present studies investigate whether this *goal*-*gradient* can also be observed in chance determined situations, situations in which there is a desired end-state (i.e., winning) but in which increased effort investment does not support goal-attainment. Three studies provide consistent evidence for the goal-gradient in chance determined situations. We show that participants (in the lab and in a TV game show) invest more effort into goal-directed behavior the closer they get to the end of the game. The moderation of expectancy and value was, however, modest. Interestingly, participants’ self-reports suggest that their dynamic changes in behavior were unintentional and perceived as non-instrumental. Findings are related to theories of goal pursuit and illusory control, and contrasted to the principle of resource conservation, according to which such behavior should not occur.

## Introduction

People often behave irrationally in chance situations. For example, people playing the lottery tend to choose meaningful number sequences (e.g., birthdates) in the hope of increasing their chance of winning (see Goodman and Irwin 2006). Similarly, people might spin the wheel faster, or shake the dice longer when the stakes are higher. These often observed *irrational* behaviors illustrate the diverse ways in which people deal with chance and uncertainty (Kahneman and Tversky 1979; Langer 1975), and pose the question whether those behaviors are systematic or functional in any way. Unfortunately, not much is known about the rules and motives behind such behavior. The present studies address this gap and suggest that behavior in chance situations can best be understood as automated goal-directed action (Bargh 1994). We propose that people pursue the positive end-state of winning a game of chance in a similar fashion as they would pursue other (more controllable) goals—even if this, rationally speaking, does not pay off.

## (Irrational) Goal pursuit

Once a goal is activated (explicitly or implicitly) several self-regulatory processes are initiated to energize and direct behavior toward goal attainment (Bargh et al. 2001; Elliot 2006; Förster and Jostmann 2012; Förster et al. 2007; Gollwitzer and Moskowitz 1996). One of the more basic self-regulatory processes supporting goal attainment is captured in the goal-gradient hypothesis, according to which motivational intensity increases as distance to the goal decreases (Brown 1948; Lewin 1935; Miller 1944). This makes sense given that with each step toward the goal one’s personal investment grows, making the goal more valuable and important to attain. As a consequence, people engage more vigorously in goal-directed behavior to ensure goal attainment. The goal-gradient occurs in animals (rats run faster the closer they get to food reward) and humans (consumers purchase more coffee the closer they get to completing their coffee stamp-card, Kivitz et al. 2006).

Importantly, how much one invests during goal pursuit depends on whether increased effort (a proxy of motivational intensity) is justified either by its instrumentality for goal attainment, and by the overall value of the goal (see expectancy-value model of behavior; Bijleveld et al. 2012; Förster et al. 2007; Liberman and Förster 2008; Silvestrini and Gendolla 2013). Instrumentality is low when the overall expectancy of success is low (e.g., when goals are unrealistic), or when one feels that one’s effort is not functional (i.e., when it does not increase the likelihood of goal attainment). This assumption is in line with fundamental principles of behavior regulation (principle of resource conservation, Hull 1943), according to which the human organism expends energy only when necessary and when it yields a return (Brehm and Self 1989; Gendolla and Wright 2009). Instrumentality is therefore considered one of the central determinants of motivational intensity, and thus also of effort investment. In chance situations, engaging more vigorously in the behavioral action does not increase the likelihood of goal attainment, so instrumentality is zero. Therefore, we should not observe any dynamic changes in the motivational intensity of goal-directed action in chance situations.

But as the above examples illustrate, people readily invest non-instrumental effort in chance situations, thereby violating the principle of resource conservation. The principle would still be met if we assumed that they actually believed that the increased effort was instrumental. While this might indeed be the case with a few individuals, most people (including many gamblers, Davis et al. 2000; Sevigny and Ledouceur 2003) know that more effort investment during a game of chance will not increase the objective likelihood of success. We argue that the most parsimonious explanation of why people nevertheless engage in such non-instrumental effort investment is because those situations involve a “desired state that one aims to attain” (i.e., a goal; p. 130, Custers and Aarts 2005; see also Gollwitzer and Moskowitz 1996). Once the goal is triggered, it is followed by the instant initiation of basic self-regulatory and motivational processes aimed at securing goal attainment (Förster and Jostmann 2012). If that is correct, then we should observe dynamic changes of motivational intensity as a function of the strength of the goal (distance, value, and expectancy). Also, then the mere activation of a goal is strong enough to initiate active goal pursuit despite a possible imbalance between investment and return.

## The present studies

Based on the last argument, we propose that in a game of chance the mere presence of the goal of winning initiates goal striving behavior characterized by the same dynamic changes in motivational intensity as observed in controllable skill-oriented situations (see also Langer 1975). In all studies participants play a game of chance, in which motivational intensity of their behavior is entirely non-instrumental, as it will have no impact on objective success probability. We operationalize motivational intensity as active effort investment during the requested game-specific action.^1^ Specifically, we predict that participants’ effort investment will vary as a function of distance to the goal (goal-gradient; Studies 1–3), and as a function of their expectation of success (expectancy, Study 2), and prize money (value, Study 3). On an exploratory note, we also investigate whether active goal pursuit in chance situations is mainly an automated response to goal activation, or whether it might actually be instrumental for a more general need (or goal); i.e., increase perceptions of (illusory) control over the outcome (Labroo and Kim 2009; Langer 1975; Preston and Wegner 2009; Wegner and Sparrow 2004).

## Study 1

In Study 1 we investigated goal striving behavior in a real-life chance situation: a daily broadcasted Dutch game show. We predicted increased effort investment (i.e., more movements and thus longer drawing times) toward the end of the game. We also predicted that once drawing the ball ceases being conducive to the goal of winning, level of effort investment would drop.

### Method

#### Participants

The ball drawing behavior of 54 teams of two (*N* = 108; 77 women) was coded, all taking part in the finals of the Dutch daily television game show ‘Lingo’ between October 2009 and May 2010.^2^ Depending on the analysis, different sub-samples are used.

#### Procedure

In the final phase of the game, one team plays to win € 5000. To win, they have to draw those numbered balls out of a bowl (containing 16 numbered balls in total), which would form a straight line in a Bingo matrix. The amount of balls they may draw varies as a function of their performance in the previous phase of the game. There is always one ball which results in winning the prize money, and there is one additional ball (the silver ball) which when drawn allows the team to take €2500 and stop playing. In that case, however, they still have to draw the remaining balls (for a more elaborate description of the game, please visit http://en.wikipedia.org/wiki/Lingo_(Dutch_game_show)). Team members (usually) take turns in drawing the balls. Our main dependent variable was the amount of seconds participants spent on each draw, starting from the moment the hand entered the bowl to when it was pulled out of the bowl. If either of two moments were invisible to the coder, the draw was excluded from the analysis.

### Results and discussion

Due to the irregular format of the data (each team had a different total amount of balls they could draw, *M* = 3.08, *SD* = 1.24; maximum = 6; members of the same team did not always draw the same number of balls) we conducted the following two analyses for testing the goal-gradient hypothesis. In the first analysis, we compared drawing times of the last (*M* = 3.99, *SD* = 1.85), second-to-last (*M* = 3.30, *SD* = 1.20) and third-to-last ball (*M* = 3.26, *SD* = 1.44) within each team (*n* = 29). A repeated measures ANOVA indicated that there was an overall difference between the last three draws, *F*(2, 56) = 3.49, *p* = .037, η_p_^2^ = .11). The last draw took significantly longer compared to the second-to-last draw (*p* = .039, η_p_^2^ = .14), and compared to the third-to-last draw (*p* = .038, η_p_^2^ = .15). Secondly, we compared the last two draws of each team-member (*n* = 41) and reconfirmed that their last draw (*M* = 3.88, *SD* = 1.72) took longer than their second-to-last draw (*M* = 3.17, *SD* = 1.28), *t*(40) = 2.85, *p* = .007, *d* = 0.45.

Finally, we investigated whether participants’ effort decreased once the goal of winning was not relevant anymore. To do this, we tested whether the average duration of the team’s draws *after* the silver ball (i.e., when they had chosen to stop and take the € 2500; *n* = 12) was shorter than the time it took to draw the directly preceding, silver, ball. As predicted, duration of the preceding draw (*M* = 3.48, *SD* = 1.07) was significantly longer than the average drawing time of the post-silver balls (*M* = 2.58, *SD* = 0.68), *t*(11) = 3.08, *p* = .011, *d* = 0.89.

## Study 2

Results of Study 1 provide first evidence for the operation of principles of goal pursuit in chance situations. Study 2 aimed to experimentally replicate the goal-gradient effect and to investigate the moderating role of expectancy of success. We asked participants to play a dice game in which winning a prize was purely chance-determined. While throwing the dice we monitored their hand movements. We predicted that participants would invest an increasing amount of effort (i.e., faster and/or bigger hand movements, longer throwing times) during the dice throw the closer they got to the end of the game. Moreover, we expected this gradient pattern to be stronger for participants who, towards the end of the game, had higher expectations of winning the prize.

### Method

#### Participants and design

Forty-nine students (35 women, two participants did not indicate their gender) took part in this study.^3^ All participants played a dice-game in which they were asked to throw the dice ten times to reach the highest possible sum score.^4^ The prize for throwing the highest sum score was a popular mobile digital audio player.

#### Materials and procedure

Participants provided informed consent and were introduced to the dice-game and the possibility of winning a prize. The game started with two practice throws.

During the entire game participants’ hand movements were recorded using a 3D position tracker (Polhemus Isotrak II). This tracker uses electro-magnetic fields to monitor and record the relative position of a remote sensor (attached to participants’ middle finger of their dominant hand) in the x, y and z direction at a rate of 60 samples per second. Each throw was introduced with an on screen instruction (“Please throw the dice”), and had a limited response window of 4000 ms, before and after which no data were recorded. Participants were told this sensor monitored their pulse and were asked to keep their hands still after throwing the dice. From the position data, we extracted three types of information per throw. First, the maximum velocity of each throw (in cm/s). Second, the duration of each throw (in s). And last, the largest hand movement the participant made during the throw (i.e., the maximum size of the throw, in cm).

To calculate the maximum velocity, we first calculated the velocity per direction per measurement sample (difference in position between two samples divided by the time separating them; Δp(X_N_, X_N−1_)/Δt(X_N_, X_N−1_). Then, we merged the velocity estimates across the three directions per sample [sqrt((vX)^2^ + (vY)^2^ + (vZ)^2^)], and selected the maximum value. The duration of a throw was defined as the total amount of time the hand was moving. To estimate that, we first calculated the standard deviation (SD) of the velocity of each individual throw (based on the merged estimates). All samples in which the velocity exceeded the baseline (velocity = 0) by one SD were accumulated and converted into a temporal estimate by multiplying it with time per sample (1 s/60 samples). Finally, to determine the maximum size of each throw, we computed all distances between all positions (240 samples) using the 3D Pythagoras formula, sqrt((X_i_ − X_j_)^2^ + (Y_i_ − Y_j_)^2^ + (Z_i_ − Z_j_)^2^), and selected the largest of all distances.

The outcome of each throw was recorded by the experimenter. After ten throws participants learned about their final score, and were asked to rate the likelihood of winning the prize (1 = *very unlikely*, 9 = *very likely*). Finally they were asked to provide some demographic information, were debriefed and dismissed.

### Results and discussion

We excluded one participant who did not adhere to the procedure. The remaining 48 participants (*M*_age_ = 22.72, *SD* = 6.05; 35 women) were included in the analyses. As in Study 1, we tested the goal-gradient hypothesis by zooming in on the last three throws (8, 9, 10). If motivation increased as a function of the distance to the goal it should be most visible towards the end of the game.

#### Maximum velocity

We first tested whether the velocity with which participants performed the throw increased across the last three throws. A repeated measures ANOVA revealed no effect, *F*(1.60, 79.48) = 2.17, *p* = .129, η_p_^2^ = .04 (Greenhouse-Geisser correction for non-sphericity). Simple contrasts indicated that the maximal velocity in the last, 10th throw (*M* = 102.09, *SD* = 57.55) was not significantly larger than in the 9th throw (*M* = 94.36, *SD* = 48.04, *p* = .102, η_p_^2^ = .06) or in the 8th throw (*M* = 92.16, *SD* = 50.83, *p* = .103, η_p_^2^ = .06). To test the moderating role of expectancies, we included their sum score after the 8th throw as an additional continuous factor. That sum-score represented a relatively objective measure of their expectation of winning and was related to their subjective expectancy (assessed after the game, *r* = .37, *p* = .010). Neither this analysis, nor a median split (at 28) analysis on the high and low expectation group separately, revealed any effects.

#### Duration

We then tested whether the duration of participants’ throws increased across the last three throws. Another repeated measures ANOVA revealed a main effect of the repeated factor, *F*(2, 94) = 6.32, *p* = .003, η_p_^2^ = .12. Participants spent more time throwing the last dice (*M* = 1.14, *SD* = 0.46) compared to the 9th dice (*M* = 0.96, *SD* = 0.44, *p* = .006, η_p_^2^ = .15) and to the 8th dice (*M* = 0.96, *SD* = 0.44, *p* = .003, η_p_^2^ = .17). Adding the sum score after the 8th throw as a continuous factor only showed an additional main effect of score, *F*(1, 44) = 6.56, *p* = .014, η_p_^2^ = .13. The higher participants sum score after the 8th throw, the longer their average duration of the last three throws, *r* = .36, *p* = .014. When splitting the group on the median sum score and running the analysis for both groups separately, only the group that had high expectations of winning shows the main effect of the repeated factor (*p* = .014, η_p_^2^ = .16; low expectation group *p* = .202).

#### Maximum size

Finally, we tested whether participants’ hands traversed larger distances the closer they got to the end of the game. We ran a similar repeated measures ANOVA and obtained a significant effect of the repeated factor, *F*(1.62, 76.33) = 10.63, *p* < .001, η_p_^2^ = .18 (Greenhouse-Geisser correction for non-sphericity). Simple contrasts showed that the maximum size of hand movements was larger in the last (*M* = 20.33, *SD* = 13.25) compared to the 9th throw (*M* = 15.74, *SD* = 11.16, *p* = .002, η_p_^2^ = .18), and compared to the 8th throw (*M* = 15.06, *SD* = 9.75, *p* < .001, η_p_^2^ = .25). Adding their sum score after the 8th throw as an additional continuous factor, or splitting the group in high and low expectation subsamples, did not affect their pattern of throwing behavior.

#### Exploratory analyses

We computed correlations between participants’ hand movements and their explicit chance ratings in order to test whether increased effort investment would lead to increased illusory control. Results suggest that this is not the case. Instead, their explicit chance ratings were largely related to their actual final sum score (*r* = .39, *p* = .006).

Finally, we wanted to see whether participants’ level of superstitious beliefs or desire for control could explain the obtained goal-gradient effect. For that purpose, participants filled in two individual difference measures at the very beginning of the experimental session. The first measure was the superstitious belief questionnaire (adapted from Wiseman and Watt 2004). Participants rated 14 statements (e.g., “I believe that the number 13 is unlucky”; α = .84) on Likert-type scale ranging from 1 (*completely not true*) to 7 (*completely true*). The second measure was the Desirability of Control scale (translated into Dutch, adapted from Burger and Cooper 1979); participants rated 17 statements (e.g., “I enjoy making my own decisions”; α = .48) on the same scale. Scores were averaged, with higher scores indicating stronger superstitious beliefs and desire for control.

Scores on the superstitious beliefs questionnaire indicated that our sample did not hold very strong superstitious beliefs (*M* = 2.00, *SD* = 0.83). Moreover, superstitious beliefs were no moderator in any of the reported results. Due to the low reliability of the second scale, we did not include it in the analysis.

## Study 3

Study 3 explored the moderating role of value in the goal-gradient effect, by varying the amount of prize money participants could win. We predicted that the goal-gradient would be steeper in the condition with higher prize money.

### Method

#### Participants

Ninety-three female students took part in the present study. Participants were randomly assigned to one of four reward conditions.^5^

#### Design and procedure

After having provided informed consent, participants were asked to throw a dice six times using a regular dice cup. There were two versions of the game: in version 1 the person who would throw the *highest* sum score would win the prize (condition *high score*); in version 2 the person who threw the *lowest* sum score of all participants would win the prize (condition *low score*). The version participants would play was determined by their summed score after three throws (>9 → high score, = <9 → low score). This way, we could reduce differences in participants’ expectancy of success. Participants believed that the assignment to version was random and after three throws the experimenter told them which version they played. Besides the version of the game, we also varied the amount of money participants could win (€ 0 vs. € 10 vs. € 50 vs. € 100). Participants knew the prize money from the beginning. This created a 2 (version of game) × 4 (financial reward) between-subject design.

The experimenter recorded the outcome of each throw. As dependent measures we timed the duration of their throw, and counted the number of times they shook the dice cup. This coding was done after data collection and was based on video-footage of the game (participants were filmed unobtrusively throughout the game). As in Study 1 and 2, greater duration and number of movements were interpreted as indicators of higher effort investment. At the end of the game, we asked participants to estimate their “chance” and “desire” of winning, and we asked whether they think they might have increased their chance through throwing “harder” or “softer” (all questions were answered on a 7-point Likert scale with higher numbers indicating a more endorsement).

### Results and discussion

We excluded nine participants because they did not follow instructions, or because they were accidently assigned to the wrong version of the game, or because their data was missing. The final sample consisted of 84 female participants (*M*_age_ = 20.30, *SD* = 1.93; 36 playing the low version).

We first checked whether the reward manipulation led to differences in participants’ reported desire to win. Whereas there was no difference in reported desire to win between the two lowest (*M*_0_ = 4.78, *SD* = 1.90; *M*_10_ = 5.41, *SD* = 1.33; *t*(38) = −1.23, *p* = .225, *d* = 0.40), and two highest reward conditions (*M*_50_ = 5.87, *SD* = 1.36; *M*_100_ = 6.00, *SD* = 1.45; *t*(42) = −.31, *p* = .759, *d* = 0.09), the two highest conditions together reported a significantly stronger desire to win compared to the two lowest conditions taken together (*M*_high_ = 5.93, *SD* = 1.39; *M*_low_ = 5.13, *SD* = 1.62; *t*(82) = −2.46, *p* = .016, *d* = 0.53). We therefore collapsed the two lowest financial reward conditions (€ 0 and € 10) and the two highest financial reward conditions (€ 50 and € 100) to create a *low* and *high reward* condition.

#### Duration of movements

As previously, we zoomed in on the last three throws. Version of the game did not significantly influence the reported results, so it was dropped from the statistical design. To test our hypotheses, we conducted a repeated measures ANOVA with the average duration of the 4th, 5th and 6th (last) throw as the repeated factor and reward condition (low vs. high) as between-subject variable. There was a significant main effect of the repeated factor, *F*(1.55, 126.96) = 3.92, *p* = .032, η_p_^2^ = .05 (Greenhouse-Geisser correction for non-sphericity). Simple contrasts confirmed that participants spent more time on the 6th compared to the 5th throw (*p* = .003, η_p_^2^ = .10; no difference between the 6th and the 4th, *p* = .725, see Table 1 for descriptives). The interaction with reward condition did, however, not reach significance (*F* < 1). Due to our specific hypothesis, we looked at simple effects which revealed that only in the high reward condition, the 6th throw took longer than the 5th (*p* = .007, η_p_^2^ = .16; low reward condition *p* = .185).

**Table 1:** Mean (SD) duration and number of movements of the 4th, 5th and 6th (last) throw

		Throw		
		4	5	6
Reward	Variable	*M* (*SD*)	*M* (*SD*)	*M* (*SD*)
Overall (*N* = 84)				
	Duration	1.66 (0.63)	1.50 (0.54)	1.63 (0.67)
	Number	4.77 (2.41)	4.14 (1.96)	4.73 (2.74)
High (*n* = 44)				
	Duration	1.71 (0.71)	1.48 (0.58)	1.67 (0.84)
	Number	4.84 (2.78)	3.95 (1.87)	4.77 (3.03)
Low (*n* = 40)				
	Duration	1.61 (0.53)	1.52 (0.49)	1.60 (0.42)
	Number	4.70 (1.96)	4.35 (2.07)	4.68 (2.42)

#### Number of movements

The same analyses were conducted for the number of movements participants made during each throw. Closely mirroring the above results, there was a main effect of the repeated factor, *F*(1.71, 140.59) = 4.19, *p* = .022, η_p_^2^ = .05 (Greenhouse-Geisser correction for non-sphericity). Again, participants made more movements during the 6th compared to the 5th throw (*p* = .003, η_p_^2^ = .10; no difference between the 6th and the 4th, *p* = .866, see Table 1 for descriptives). Again, the interaction with reward condition did not reach significance (*F* < 1). As above, the contrast between the 6th and 5th throw was, however, only significant in the high reward condition (*p* = .005, η_p_^2^ = .17; low reward condition *p* = .217).

Together those results provide additional evidence for the goal-gradient hypothesis, support for the moderating role of value was, however, weak. Unlike in Study 1 and 2, we only found an increase in effort investment between the last and second-to-last throw, but not between the last and the third-to-last throw. The lack of linearity could possibly be explained by the observation that some participants asked additional questions concerning the version-of-game assignment during their 4th throw, thereby artificially inflating the measures.

#### Exploratory analyses

To investigate whether participants’ increased effort investment towards the end of the game resulted in increased illusion of control, we correlated their behavioral measures with their explicit chance ratings. As in Study 2, there was no relation between participants’ behavior during the game and their explicit chance ratings after the game. Instead, participants’ explicit chance ratings were related to their final sum score (high version: *r* = .41, *p* = .003; low version: *r* = −.26, *p* = .122).

We also checked whether or not participants had the sense that adjusting one’s manner of throwing (harder vs. softer) would have had an influence on the results. Similar to Study 2, participants did not endorse such a superstitious belief (*M*_harder_ = 1.33, *SD* = 0.76; *M*_softer_ = 1.51, *SD* = 1.24), nor did it influence any of the above effects.

## General discussion

The present studies investigated seemingly irrational effort investments during games of chance by applying a goal pursuit perspective. Results of three studies consistently showed that people invest more effort into goal-directed action (i.e., more and larger hand movements, longer durations) the closer they get to the end of the game. That behavioral pattern resembles a goal-gradient, which is defined as increased motivational intensity as the distance to the goal decreases (Brown 1948; Lewin 1935). We therefore suggest that the non-instrumental effort investments observed in our studies might be due to the mere presence of the goal of winning, which in turn initiates a set of self-regulatory processes facilitating goal attainment. That conclusion is further corroborated by the finding that increased effort during goal-directed action did not increase perceptions of control over the outcome, neither did it vary as a function of participants’ desire for control or superstitious thinking. Moreover, explicit reports strongly suggest that those dynamic changes in effort were unintentional (or denied). In line with recent theorizing on automatic self-regulation (e.g., Förster and Jostmann 2012), we therefore conclude that the goal-gradient pattern of our participants’ behavior can best be explained as automatically following from goal activation.

Our prediction that participants’ goal-directed action would only follow a gradient shape if the expectancy (Study 2) and value (Study 3) of winning is high was, however, not consistently confirmed. Participants’ behavior resembled a goal-gradient even when their expectancy of success was relatively low, and even when the prize they could win was less valuable. That null finding could be due to our specific operationalization of expectancy and value. First, in Study 2 we used the score at the 8th throw as an indicator of expectancy of success. Possibly participants did not use that specific outcome to guide their subjective expectancy—even if that was their safest guess. Also, given that the score of other participants was unknown at the time of playing, their score after the 8th throw might have remained relatively meaningless to them. Subjective likelihood of success may have to be extremely low or absent in order to find the predicted effect. Nevertheless, additional analyses showed that the higher their sum score after the 8th throw, the longer they spent on the last three throws more generally. Second, our value manipulation in Study 3 might not have been strong enough. Though the amount of the prize money affected participants’ explicit desire to win, it is possible that subtle variations in expectancy interacted with their subjective value estimates. Future studies should assess or control those variables more reliably, through creating more distinct categories.

Taken together, we interpret our findings as support of an automatic goal pursuit perspective on behavior in chance situations. Our studies have shown that the attraction that emanates from positive end-states, such as winning a game, can be strong enough to engage people in non-instrumental effort investment. That demonstrates that positive goals can gain such motivational relevance that even fundamental principles of human behavior, such as the principle of resource conservation, are challenged. There is, however, a way of reconciling this apparent inconsistency. First, it could be that the effort participants invested was so modest that its perceived cost approached zero. If costs are zero than ‘no return’ is no loss and any return a gain. That way it may even be more adaptive to exert the extra effort (for similar argumentation see also error management theory, Haselton and Buss 2000). Second, from previous research we know that people’s chance estimates are blurred in situations of uncertainty (e.g., Arrow 1982; Langer 1975). Even if one can report that a situation is completely chance determined, it may not be reflected in one’s judgment or behavior (see also Sevigny and Ledouceur 2003). That is especially the case when chance situations contain skill-related elements (e.g., possibility to engage actively; Langer 1975), or—as our studies demonstrate—when they imply an attractive goal.

Though we are convinced that the automated goal pursuit account offers the most parsimonious explanation for our data, two alternative explanations should be mentioned. For example, one could argue that longer drawing times serve to delay possible disappointment (i.e., losing). Such an emotion regulation strategy might sound plausible but is neither supported by Study 1 (participants are quicker to draw the post-silver balls, even though the possibility of disappointment is even more pronounced here), nor by research on the delay of negative outcomes (Loewenstein 1987; Lovallo and Kahneman 2000). Conversely, one might argue that increased drawing times serve to prolong the state of positive anticipation. Although that seems more in line with research on the delay (i.e., savoring) of positive uncertain outcomes (Lovallo and Kahneman 2000), such research would only predict effects on the temporal, but not on the more direct effort measures (i.e., more and larger movements). In fact, if people were trying to delay the outcome because they want to savor the prospect of winning, we should observe invariable or even *less* vigorous goal-directed behavior towards the end of the game. Given our pattern of results, we conclude that this investment most likely indicates goal pursuit rather than only emotion regulation.

Besides the goal-gradient, goal pursuit has a number of additional characteristics relevant for understanding why and how people engage in (irrational) behaviors in chance situations. A goal pursuit analysis could therefore provide us with new insights as well as potential intervention strategies in areas such as pathological gambling. For example, when focused on a goal (e.g., winning a dice game) goal-relevant or goal-conducive events (e.g., one good throw) will receive more attention and more positive evaluations compared to goal-irrelevant or goal-obstructing events (e.g., one bad throw; see Fishbach and Ferguson 2007). This could distort (i.e., inflate) the assessment of one’s personal chance of achieving the goal. The consequences of such a mechanism (e.g., continue playing or increasing stakes under the exaggerated impression of high chances of winning) can be especially dramatic in the gambling context. Moreover, from goal theories we know that while one goal is activated (e.g., winning the gamble) competing goals (e.g., reducing gambling for the sake of saving money) are inhibited (see Stroebe et al. 2013). Whereas such mechanisms generally support goal attainment, they can, for example, also contribute to the slippery slope of gambling. Raising awareness for those automatic mechanisms could prove useful when trying to reduce the risky behavioral patterns observed in gambling.

## Conclusion

Three studies consistently showed what appears to be goal-striving behavior in chance situations. This finding is important because it provides strong evidence for an automated goal pursuit account (e.g., Bargh 1994). While automated goal pursuit is undoubtedly beneficial in situations in which increased goal-directed action increases realistic success probabilities, our studies suggest that it even occurs in situations in which success probabilities are unaffected by variations in goal-directed action. The present studies therefore emphasize the flexibility with which we motivationally respond to and interact with the world—despite the risk of behaving irrationally.

## Footnotes

1 Pilot data (*N* = 101) suggested that people associate motivation and success with more ‘active’ adjectives (e.g., forceful, fast) rather than more ‘passive’ adjectives (e.g., soft, careful).

2 At the time of data collection we stopped when no more episodes was available. Eventually the sample size appeared to be appropriate for most of our analyses. A power analysis for three repeated measures with *f* = .25, α = .05, power = .80 requested minimally 28 participants.

3 At the time of data collection, we followed the’at least 20 per cell’ stopping rule. Eventually the sample size appeared to be appropriate for our main analyses. A power analysis for three repeated measures and two groups with *f* = .25, α = .05, power = .80 requested minimally 28 participants.

4 Participants also played a different dice game (in counterbalanced order), which will not be reported in the present paper. Order of tasks did not have a significant effect in any of the analyses.

5 See footnote 3.
